# Magnetoreceptory Function of European Robin Retina: Electrophysiological and Morphological Non-Homogeneity

**DOI:** 10.3390/cells11193056

**Published:** 2022-09-29

**Authors:** Alexander Yu. Rotov, Arsenii A. Goriachenkov, Roman V. Cherbunin, Michael L. Firsov, Nikita Chernetsov, Luba A. Astakhova

**Affiliations:** 1 Laboratory of Evolution of the Sense Organs, Sechenov Institute of Evolutionary Physiology and Biochemistry RAS, 194223 St. Petersburg, Russia; 2 Spin Optics Laboratory, Physics Faculty, St. Petersburg State University, 198504 St. Petersburg, Russia; 3 Department of Vertebrate Zoology, Biological Faculty, St. Petersburg State University, 199034 St. Petersburg, Russia; 4 Ornithology Lab, Zoological Institute RAS, 199034 St. Petersburg, Russia

**Keywords:** retina, birds, magnetic compass, magnetoreception, cones, oil droplets

## Abstract

The avian magnetic compass allows orientation during migration and is shown to function properly under short-wavelength but not long-wavelength visible light. Therefore, the magnetoreceptive system is assumed to be light- and wavelength-dependent and localized in the retina of the eye. Putative candidates for the role of primary magnetosensory molecules are the cryptochromes that are known to be expressed in the avian retina and must be able to interact with phototransduction proteins. Previously, we reported that in migratory birds change in magnetic field direction induces significant effects on electroretinogram amplitude in response to blue flashes, and such an effect was observed only in the nasal quadrant of the retina. Here, we report new electroretinographic, microscopic and microspectrophotometric data on European robins, confirming the magnetosensitivity of the retinal nasal quadrant after applying the background illumination. We hypothesized that magnetoreceptive distinction of this region may be related to its morphology and analyzed the retinal distribution and optical properties of oil droplets, the filtering structures within cones. We found that the nasal quadrant contains double cones with the most intensely colorized oil droplets compared to the rest of the retina, which may be related to its magnetosensory function.

## 1. Introduction

Birds use the Earth’s magnetic field (MF) for orientation and navigation. These behavioral tasks require compass and positional information, respectively, and birds can achieve both from the geomagnetic field [1,2]. Some indirect evidence suggests that the avian magnetic compass is light-sensitive, moreover, it is wavelength-dependent: migratory birds of different species show season-specific orientation in behavioral experiments under short-wavelength (near ultraviolet, blue and green) illumination, but become disoriented under long-wavelength (yellow and red) light [3,4,5,6]. It was hypothesized that the magnetoreceptive system is localized in the retina of the eye and somehow coexists with photoreception, using photons of short-wavelength light to activate the radical reaction sensitive to the direction of an external magnetic field [7]. The only known biological molecules found in vertebrates that can serve as primary sensory molecules for MF perception according to the radical reaction mechanism are the cryptochromes [8,9]. These photosensitive proteins are known to participate in circadian regulation, but at least three types of cryptochromes, Cry1a [10], Cry1b [11] and Cry4 [12], were shown to be expressed outside the nucleus in the cells of the avian retina, which makes them promising candidates for magnetic sensitivity. The “cryptochrome hypothesis” does not contradict most experimental data on avian magnetic sense; moreover, it can fairly well explain its inclination nature, sensitivity to weak oscillating MFs and wavelength dependence [13]; however, for sensitivity to oscillating MFs, see [14,15]. Recently, Cry4 was shown not only to be able to form magnetosensitive structures in vitro [16] but also to interact with cone-specific proteins known to participate in phototransduction [17,18], suggesting that its signaling pathway is colocalized with phototransduction. The localization of Cry4 in the double cones’ outer segment together with the specific morphology of this cell type makes them a promising model for a potential magnetoreceptor [13,19].

In our previous studies, we analyzed how the direction of an external MF can modulate the visual response in the whole isolated retina of pigeons, *Columba livia* [20,21], and European robins, *Erithacus rubecula* [22]. We reported that in migratory birds change in magnetic field direction induces small but statistically significant effects of the magnetic field on electroretinogram (ERG) amplitude in response to blue, but not to red, flashes. Another interesting finding was that this effect was observed only in the nasal quadrant of the European robin’s retina, implying a possible existence of a specific magnetosensitive area within the retina. Here, we report the results of the logical extension of that study with new electroretinographic, microscopic and microspectrophotometric data on European robins. We confirmed the magnetosensitivity of the retinal nasal quadrant in this species after applying the background illumination. We also propose that electrophysiological distinction of the nasal quadrant in relation to magnetosensory function may be related to its morphological features, for example, to photoreceptors’ distribution or their optical properties.

Birds possess highly developed color vision and most avian species, in addition to rods, have five types of cones containing a specific visual pigment combined with different intracellular filtering structures [23,24]. Avian cones contain oil droplets (ODs)—highly refractive spherical organelles located at the distal end of the photoreceptor inner segments [25,26]. ODs are composed of different carotenoids, giving them diverse coloration and, consequently, diverse light-filtering properties [27]. The general pattern of OD coloration among most investigated avian species is highly conservative and allows unambiguous identification of cone type [23,24]: red-sensitive (long-wavelength) single cones (LWS) contain red ODs, green-sensitive, i.e., medium-wavelength sensitive, cones (MWS) contain yellow ones, blue-sensitive, i.e., short-wavelength sensitive (SWS), cones contain clear ones and ultraviolet-sensitive (UVS) transparent and double cones contain pale ones. Therefore, one can map the particular cone type distribution throughout the whole retina by observing its OD color and size with a light microscope [28]. More detailed information about an OD’s optical properties and how they affect the spectral sensitivity of the cones can be obtained by microspectrophotometry [29,30]. To the best of our knowledge, here we characterize the distribution of cones in the European robin’s retina by quadrants and describe the ODs’ spectra of this species for the first time. Our results revealed that the nasal quadrant of the European robin’s retina contains double cones with more intensely colorized oil droplets than double cones in other quadrants, and it may be related to the unique responsiveness of the nasal quadrant to MFs. Such uncommon spectral properties are in agreement with supposed features of magnetoreceptive double cones; therefore, this increases the likelihood that there is a specific magnetically sensitive area in the avian retina.

## 2. Materials and Methods

### 2.1. Experimental Animals

Adult European robins were wild-caught during their spring migration period on the Courish Spit (Kaliningrad Region, Russia) (electrophysiology) and during the autumn migration period in Zvenigorod (Moscow Region) and then birds were moved to the lab in St Petersburg, where animals were kept in individual cages. An artificial light:dark cycle corresponding to the natural light:dark cycle at the natural location was set up to day of sacrifice. Birds were sacrificed by decapitation to eliminate the impact of injected substances on the retinal physiology. Capture, keeping and euthanasia were performed following an approved protocol (Permit-04 of 20 April 2019 issued by Bioethics Committee of the IEPHB RAS).

### 2.2. Ex Vivo Electroretinography with Magnetic Field Modulation

**Figure 1:**
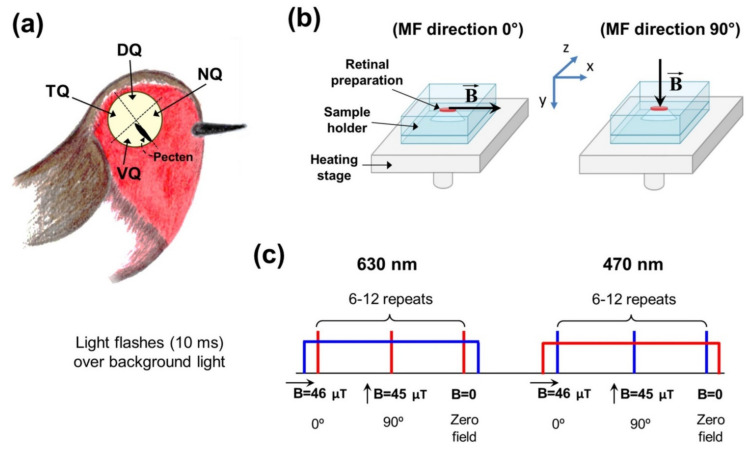
(**a**) Schematic representation of the bird’s retina dissection used for obtaining samples for electrophysiological, microscopic and microspectrophotomentric examinations. NQ, VQ, TQ and DQ—nasal, ventral, temporal and dorsal quadrants, respectively. (**b**) Representation of two directions of application of MF to the retinal samples. A sample of every quadrant of the retina was put into the sample holder in a horizontal plane and, during the experiment, the magnetic vector was directed either parallel or perpendicular to the plane of the retina. The *xyz* coordinate system shows the orientation of the probe of the magnetometer. (**c**) Schematic diagram of the experimental protocol. In the present study, two sorts of light stimulus under changing magnetic field were tested: brief red flashes under continuous dim blue background light and brief blue flashes under continuous dim red background light. Our protocol involved using three settings of MF: first with an angle between MF lines and the plane of the retina of 90°, second with angle 0° and third with the zero field, all three repeated 6–12 times. MF—magnetic field.

Our experimental setup and procedure of ex vivo ERG recording from isolated avian retinas were described in detail in a previous study [22]. Briefly, before decapitation birds were dark-adapted for approximately one hour; after decapitation, their eyes were enucleated and eyecups were prepared. Every eyecup was divided into the nasal ventral temporal and dorsal quadrants (pecten served for orientation, see Figure 1a) and retinal fragments were extracted. ERG recordings were performed for every fragment separately and both left and right eyes were used. All these steps were performed using dim red light (with λ > 600 nm). For tissue preparation and for perfusion during ERG experiments, the Ames medium (A1420, Sigma–Aldrich, St Louis, MO, USA), supplemented with (in mg per liter): KCl 144, MgCl_2_ 167, NaHCO_3_ 2100 and Glucose 3419 (all purchased from Sigma-Aldrich, St Louis, MO, USA) was used. During perfusion, this medium was continuously heated to 37 °C and bubbled with a mixture of 95% O_2_/5% CO_2_.

The responses to light were recorded in a closed-type specimen holder where an isolated retina was placed photoreceptors downward. The signals from the retina were amplified and low-pass filtered at 1000 Hz by a differential amplifier (DAM50, WPI, Sarasota, FL, USA) and captured at a sampling interval of 2 ms by the data acquisition board. For controlling the external MF around the isolated retina during photoresponse recording, a custom-built system consisting of three pairs of orthogonal Helmholtz coils was used. The diameter of coils was approximately 30 cm. MF settings were controlled by a laboratory PC through two 12-bit D/A converters. The coil-generated MF compensated for the Earth and laboratory fields and created a magnetic vector oriented in the vertical plane in a controlled manner (see Figure 1b) with amplitude 45–46 µT (close to Earth MF magnitude). Thus, during the experiment, coils generated an MF for compensation for the ambient MF existing in the lab and creation of an MF arbitrarily oriented in the vertical plane. This configuration allows establishing the vertical or horizontal direction of the MF with an amplitude of approximately 45 μT, i.e., perpendicular or parallel to the plane of the retina. To avoid induction artefacts caused by discrete MF rotation by 90° ([31]), our protocol included a 1s delay between MF rotation and light stimulus application. It should be sufficient to separate the induction artifact and light response. The field direction switching time was less than 1 ms. Additionally, to reduce the voltage induced at the input of the amplifier by rapidly changing magnetic field, the wires leading to the positive and negative electrodes were placed as close to each other as possible, since the induced effect is proportional to the area between the wires. The parameters of MF were monitored and registered during the experiment by a custom-made three-coordinate magnetometer based on an eCompass LS303DHL module (STMicroelectronics, Geneva, Switzerland) located under the stage for the sample holder. This magnetometer measures the MF with accuracy ±1 µT. To eliminate possible signal disturbance from ferromagnetic components of the setup, the perfusion chamber with the retina sample and the Helmholtz coils were located at a distance of 1.5–2 m from all iron-containing devices. Additionally, an aluminum box surrounded the sample holder with the retina for protection from the external electromagnetic interference. Integration of the noise density in a range of 0.1–10 MHz gives the total amplitude of the alternating MF near the retina sample approximately B = 8 nT (slightly less than the sensitivity threshold of a hypothetical magnetic compass to an oscillating MF [32]).

The light stimulation system included high-output light-emitting diodes (LEDs). Light intensity for stimulation was adjusted by LED current and the set of neutral density filters. The preparations were tested with brief flashes (10 ms) of red or blue LEDs (λ_max_ = 630 or 470 nm) combined with continuous background illumination from the same LEDs (Figure 1c). Blue and red flash intensities were chosen independently for each retina sample to excite almost equal electrical responses. For emission spectra of the LEDs used in the present study, see Supplementary Figure S1. Three different settings of MF were used in the protocol: first, light stimulus under an MF of 46 µT, with an angle between the magnetic vector and the plane of the retina of 90°, second, light stimulus under an MF of 45 µT and with this angle equal to 0° and, third, light stimulus under zero MF. For each intensity and each light (blue or red), this sequence was repeated 6 to 12 times. For all light stimuli in the sequence, there was a 1 s delay between changing the MF setting and light flash. For controlling stimulus intensity and timing and for data acquisition, National Instruments hardware and LabView 16.0 software (National Instruments, Austin, TX, USA) were used.

### 2.3. Light Microscopy

For light microscopy, the eyecups were prepared the same way as for electroretinographic recordings. The eyes of the same animal were shared between microspectrophotometric measurements and light microscopic examination: the first eyecup (either right or left between individuals) was prepared for retinal whole-mount preparations of fresh retina and the second one was divided into four separate quadrants used for microspectrophotometry or light microscopy. The extracted retina (whole-mount or excised quadrant) was placed on a non-gelatinized glass slide in a few drops of PBS, photoreceptor side up. In the case of whole-mount preparations, four radial incisions were made around their circumference to flatten them, and any remaining pecten tissue was gently dissected from the border between ventral and nasal parts of the retina. To support an intact coverslip laid on top of the preparation, we used “legs” made of fragments of another coverslip (with thickness approx. 0.2 mm). This approach was adopted from a study by Kram et al. [33] and allows keeping the mosaic reasonably intact. Images of the retina were taken at 40× or 20× magnification at the level of the oil droplets in two different planes of focus using an Olympus BH-2 light microscope (Tokyo, Japan) equipped with a Canon E05 1000D digital camera (Tokyo, Japan). Additional images were captured with a purple glass filter (400 nm cut-off wavelength) that facilitated distinguishing between truly transparent and slightly colored ODs. Counting ODs and their size measurement for each microphotograph were performed using ImageJ software (NIH, Bethesda, MD, USA).

### 2.4. Microspectrophotometry of Oil Droplets

Absorbance spectra of ODs were recorded using a microspectrophotometer—the single-beam spectrophotometer combined with microscope optics allowed measurement of micron-sized objects. The design of the instrument as well as the procedures for sample preparation and recording have been described in detail earlier [30,34]. Samples for determination of ODs’ spectral characteristics were prepared by chopping a small piece of the retina from the quadrant of interest in a drop of PBS supplemented with 40% sucrose. The preparation was placed between two coverslips sealed at the edges and fixed on the microscope preparative table. The additional retinal preparations were stored at 4 °C until use to reduce tissue degradation. Recordings were performed from the ODs of cones attached to the border of small retinal pieces. The absorbance spectra were recorded with a square-shaped measuring beam not exceeding the borders of the OD, in the wavelength range 330–760 nm with 1 nm discretization. Since ODs act as cut-off filters, they were characterized according to their “cut-off” wavelength (λ_cut_), which is defined as the wavelength on the intercept of the maximum absorbance value by the line tangent to the spectrum slope at its half maximum [35].

### 2.5. Data Analysis

To determine the potential effects of external MF direction on the ERG responses, the amplitudes of their main components (a- and b-wave) recorded under the angle between the MF vector and the plane of the retina of 90° and 0° were compared. We normalized amplitudes of all responses to the corresponding values for the responses recorded under a zero MF. Further, we analyzed the kinetics changes in ERG components by making a comparison in time-to-peak intervals for both a- and b-waves of responses recorded under different MF directions.

Normality of all data samples was estimated by the Shapiro–Wilk test. Comparisons of time-to-peak interval data and normalized amplitudes were made using the Wilcoxon test for paired samples. When testing for the MF effect, we performed the same test four times for each time interval and amplitude parameter, applying it to the preparations of the temporal, dorsal, ventral and nasal quadrants of the retina. Therefore, to avoid false positive results we corrected the significance level of 0.05 for multiple comparisons using the Holm–Bonferroni method.

Photoreceptor distribution was analyzed by estimation of the density distributions of certain OD types and their size from the light microscope images of four retinal quadrants. For estimation of oil droplet spectra, we compared the λ_cut_ values of certain OD types between retinal quadrants. We used the Kruskal–Wallis test with post hoc Dunn test for multiple comparisons or, in case of a normal distribution, the Brown–Forsythe ANOVA test with the post hoc Dunnett test for these parameters.

All data were analyzed using Microsoft Excel 2010 (Microsoft Corp., Redmond, WA, USA) and GraphPad Prism 8 (GraphPad software, Inc., San Diego, CA, USA).

## 3. Results

### 3.1. Electroretinography under Background Light and Magnetic Field Modulation

**Figure 2:**
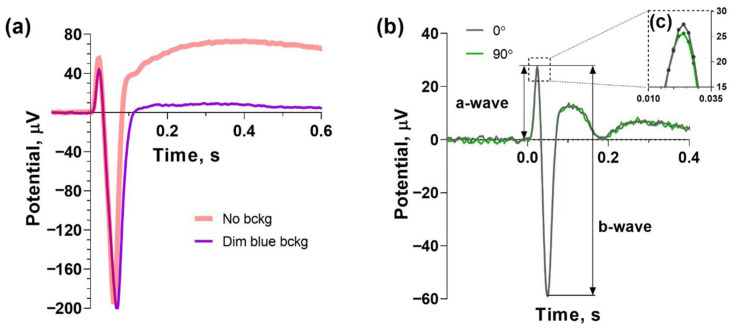
European robin’s isolated retinal ERG responses to flashes under continuous background light. (**a**) Representative pair of responses to brief 10 ms red flashes in dark-adapted state (pale red line) and under blue continuous background light (violet line) of the same retinal preparation (red flash intensity 1.3 × 10^10^ photons/mm^2^, blue continuous background intensity 1.1 × 10^10^ photons/mm^2^/s). (**b**) Representative average ERG responses of nasal quadrant preparation to the 10 ms red flashes over blue continuous background light with two different directions of magnetic field: dark gray curve—response under 0° MF direction, green curve—response under 90° magnetic field direction. Inset (**c**) shows the enlarged a-wave peak from panel (**b**). Red flash intensity 1.2 × 10^9^ photons/mm^2^, blue background light intensity is the same as for panel (**a**). MF—magnetic field.

To widen our knowledge about the modulating effect of the MF on the ERG responses in European robins in this work, we used a more complicated experimental protocol compared to our previous study [22]. We combined brief (10 ms) red or blue flashes with continuous background illumination. The schematic diagram of the experimental protocol is depicted in Figure 1c. As opposed to our previous work, where we analyzed effects of magnetic field direction on flash responses in a dark-adapted state of the retina, here we additionally applied adapting background light. Background light had the opposite color: we combined red brief flashes with blue dim background light and blue flashes with red dim background light, respectively. The intensities of blue and red background were the same for all tested preparations: 1.1 × 10^10^ photons/mm^2^/s for blue light and 4.2 × 10^11^ photons/mm^2^/s for red light. Both these backgrounds were quite dim for the retina preparations: a typical pair of responses to brief 10 ms red flashes in a dark-adapted state and under continuous blue background illumination of the same retinal preparation is shown in Figure 2a, and it can be seen that amplitudes of a- and b-waves demonstrated only a slight reduction.

After several minutes needed for stabilization of the background-adapted state of preparation, we started to apply the combination of testing brief flashes and magnetic field modulation. Typically, we recorded 6–12 repeats (18–36 responses in each set). Within one set, the sequence of first light stimulus under an MF with the direction 90° with respect to the retina plane, second stimulus under an MF with the direction 0° and the third one under a zero MF was applied. During the analysis, we averaged 6–12 responses recorded under the same MF direction to improve signal-to-noise ratio, and then two averaged responses (direction 0°, direction 90°) normalized to the amplitude of the third one (zero field) were compared (Figure 2b).

**Figure 3:**
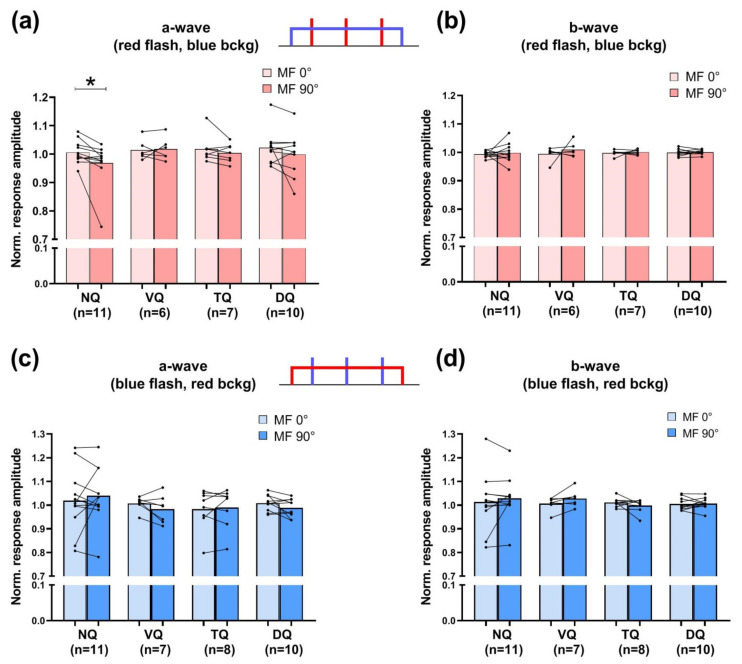
Effect of MF direction on the amplitude of the main components of ERG response, a-wave (**a**,**c**) and b-wave (**b**,**d**). Bars show the normalized a- or b-wave amplitudes obtained under certain MF directions (90° or 0° with respect to retinal plane) after stimulation with red 10 ms flashes under dim blue continuous background light (**a**,**b**) or blue 10 ms flashes under dim red continuous background light (**c**,**d**). In each panel, the results for the nasal (NQ), ventral (VQ), temporal (TQ) and dorsal (DQ) quadrants are presented separately. * Statistically significant difference according to a Wilcoxon test for paired samples with the Holm–Bonferroni correction (*p* < 0.01). Bars represent the means of corresponding samples, *n*—number of retinal preparations in each group. MF—magnetic field.

The results of the comparison of amplitudes for the a-wave and b-wave are shown in Figure 3a–d. To test for the effect of the external MF direction on the amplitude of the a- and b-waves recorded under the 0° and 90° angles, comparison of the amplitudes of these components normalized to the corresponding values of the responses recorded under a zero MF was made (the same procedure as in [22]). In our opinion, this normalization procedure allowed eliminating the confounding factor that the amplitudes of the responses are overly dependent on the sample position in the perfusion chamber and the procedure of retinal isolation. Statistical analysis shows that changing the MF direction produced a small but statistically significant effect on a-wave amplitude in responses to red flashes under blue background light only in nasal quadrants. Panels (c) and (d) in Figure 3 show amplitudes for the a-wave and b-wave in ERG responses to blue flashes under red background light and no effect of magnetic field direction is observed in this situation. As the applied flash intensities were quite high (3 × 10^8^–1.3 × 10^11^ photons/mm^2^ for red stimulus and 3.2 × 10^9^–2.9 × 10^11^ photons/mm^2^ for the blue one), our results suggest that blue light is required for magnetoreceptive function of the nasal retina but it is disturbed by too bright flashes. However, magnetoreception function is successful under continuous dim blue illumination which implies the possible mechanisms of light adaptation in magnetosensitive cells.

Additionally, we estimated the possible effects on kinetics of ERG response by comparing the time-to-peak intervals of a- and b-waves. The statistical analysis was performed the same way as for the amplitudes and we did not observe any significant differences for any type of stimulus for both ERG components (see Supplementary Figure S2). The same result we reported earlier for stimulation without any background illumination [22] indicates independence of visual response kinetics from the direction of external MF.

### 3.2. Morphological Features of Four Quadrants of European Robin’s Retina

**Figure 4:**
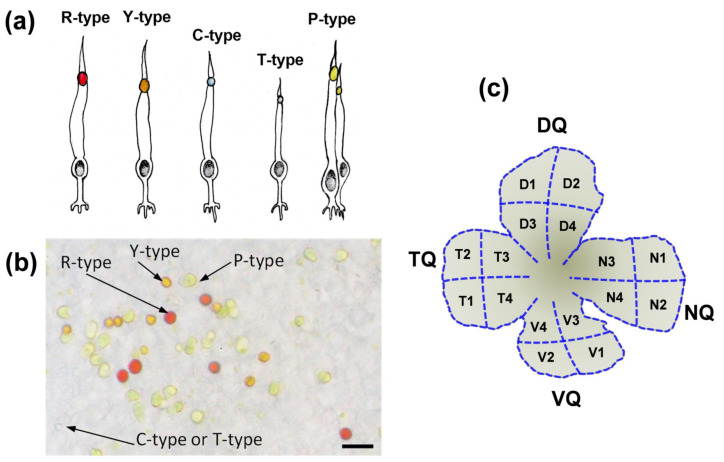
(**a**) A schematic diagram of the cone subtypes of the avian retina: long-wavelength sensitive (LWS), medium-wavelength sensitive (MWS), short-wavelength sensitive (SWS), ultraviolet sensitive (UVS) and double cones, containing R-type, Y-type, C-type, T-type and P-type ODs, respectively. (**b**) Microphotograph of a part of the European robin’s retina showing the distinctive coloration of the cone oil droplets, scale bar = 10 µm. C- and T-type ODs cannot be distinguished by the eye of the observer. (**c**) Graphical representation of the European robin’s retinal flat mount showing the 16 zones from which the areas analyzed for cone distribution were taken. Letters N, V, T and D refer to the nasal, ventral, temporal and dorsal quadrants, respectively. Numbers 1, 2 refer to the peripheral parts of the retina, while 3, 4 refer to the central ones. NQ, VQ, TQ, and DQ—nasal, ventral, temporal and dorsal quadrants, respectively; OD—oil droplet.

The retina of most studied avian species, including European robins, contains a single class of rod, a single type of double cone and four different types of single cones. A characteristic feature of the avian cones is the presence in their inner segment of the apical part of a brightly colored or colorless spherical organelle, lipid or oil droplet (see Figure 4a). Using light microscopy, it is quite easy to distinguish red (R-type), yellow (Y-type) and pale yellow (P-type) ODs but it is much more difficult to reliably distinguish transparent (T-type) and clear (C-type) ones since they both transmit most of the visible light (Figure 4b). The color type of OD is tightly coupled with the spectral type of cone visual pigment, so the R-type ODs unambiguously indicate LWS single cones, Y-type—MWS, T-type—SWS, T-type—UVS and P-type—double cones containing the same visual pigment as single LWS ones [23,24].

For analysis of distribution of R-, Y- and P-type oil droplets (reflecting the distribution of LWS, MWS single cones and double cones, respectively), we chose 16 separate areas from different zones of the retina (of either eye, left or right) and four areas for each quadrant, two from the central part and two from the periphery (see diagram in Figure 4c). First, a basic drawing was made of each retina or separate quadrant preparation (in the case of the second eye which was shared with microspectrophotometry); we suggest that such regions are quite representative. In each area, at least one picture was captured in two focal planes.

**Figure 5:**
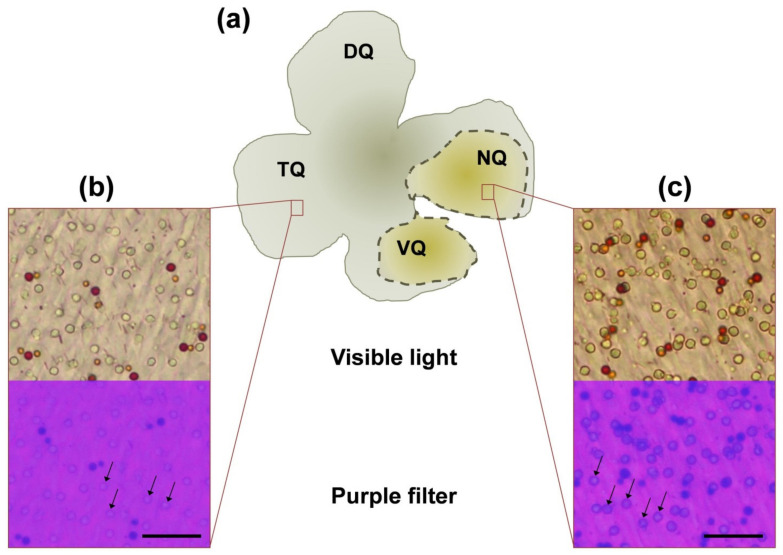
(**a**) Schematic diagram of the typical European robin’s flat-mount preparation with indication of “yellowish” areas observed in this study during gross visual examination. Boxes represent microphotographs of areas of temporal (**b**) and nasal (**c**) quadrants with typical difference in coloration of ODs of P-type. Lower parts of the boxes represent the microphotographs of the same areas made through 400 nm cut-off filter. Note that temporal P-type ODs seem transparent while nasal ones became dark because of the absorbance spectrum shift. Scale bar = 20 µm. NQ, VQ, TQ, and DQ—nasal, ventral, temporal, and dorsal quadrants, respectively; OD—oil droplet.

When analyzing said areas, we have found that P-type oil droplets exhibited different intensity of coloration in different quadrants of the European robin’s retina. Most of the nasal and some of the ventral quadrant (close to the nasal quadrant) P-type ODs had a well-marked greenish yellow color, while the same type of OD in all remaining portions of the retina had an extra pale coloration, being almost transparent. We could not quantitatively analyze this difference by analyzing light microscopic samples; however, it was visually observable by gross visual check of the whole-mount preparations that nasal and ventral parts were more yellowish than other parts. Figure 5a shows a schematic diagram of a typical European robin’s flat-mount preparation with indication of “total yellowish” areas observed in this study during gross visual examination and typical microphotographs of “yellowish” (nasal and portion of ventral quadrant) and “non-yellowish” regions of the European robin’s retina (Figure 5b,c). As the P-type ODs in most retinal areas seemed dramatically pale, both C- and T-type ODs were expected to be absolutely transparent and, consequently, indistinguishable to the observer’s eye. Indeed, we discovered rare small transparent cells that we defined as C- or T-type, but this feature did not allow us to make any calculations on SWS and UVS cone distribution separately.

**Figure 6:**
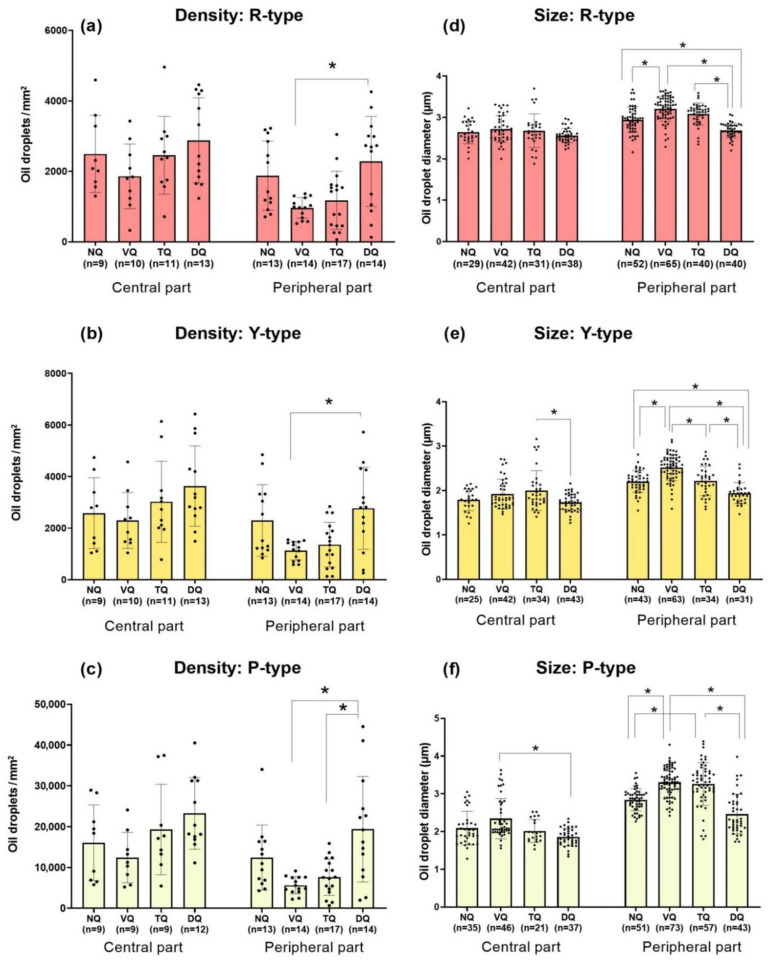
Graphical representation of the density distribution (**a**–**c**) and mean diameters (**d**–**f**) of oil droplets of certain types in the central and peripheral parts of four retinal quadrants. Data presented as mean ± SD. NQ, VQ, TQ, and DQ—nasal, ventral, temporal, and dorsal quadrants, respectively. * Statistically significant difference according to the Kruskal–Wallis test with post hoc Dunn test for multiple comparisons (*p* < 0.05). For panels (**a**–**c**), *n* is the number of analyzed retinal preparations for each area, while for panels (**d**–**f**), *n* is the total number of oil droplets measured for each area.

We estimated density of the three types of ODs (R-, Y- and P-types) in central and peripheral parts of the four quadrants (see Figure 6a–c) and found no statistically significant differences among retinal quadrants in central parts of the retina. On the other hand, in peripheral parts the highest density of all three types of ODs is observed in the dorsal quadrant with a significant difference compared to the ventral quadrant for R-type and Y-type ODs and compared to both ventral and temporal quadrants for P-type ODs. See Supplementary Figure S3 for more detailed data on the distribution of OD density for the eight directions. The prominent feature of the European robin’s retina, which is in good agreement with retinas of other passerines [30,36,37], is a characteristic mosaic of R- and Y-types ODs. The LWS and MWS single cones are distributed regularly: in most areas of the retina, they form pairs (one R-type and one Y-type) or ternaries (one R-type and two Y-types). This pattern is reflected in the ratio of densities of R- and Y-type ODs and in the percentages of the three types of OD (see Supplementary Figure S4 and Table S1). The P-type droplets are the dominant type in every area of the European robin’s retina, accounting for almost 80% of the total quantity of colored ODs.

We also estimated the ODs’ diameters throughout the European robin’s retina (Figure 6d–f). In central parts, small but statistically significant differences are observed in Y-type OD diameters between temporal and dorsal quadrants and for P-type OD diameters between ventral and dorsal quadrants. In peripheral parts, for R-type ODs there are significant differences in diameter between dorsal and all other quadrants, and between ventral and nasal quadrants (Figure 6d). For Y-type ODs in peripheral parts there are significant differences for dorsal and all other quadrants, and also for ventral compared to nasal and temporal quadrants (Figure 6e). For P-type ODs, in peripheral parts there are significant differences in OD diameters for nasal and dorsal quadrants compared to ventral and temporal ones (Figure 6f). All values for OD densities and diameters are presented in Table 1 and Table 2, respectively.

### 3.3. Spectral Characteristics of Oil Droplets from Different Quadrants of European Robin’s Retina

**Figure 7:**
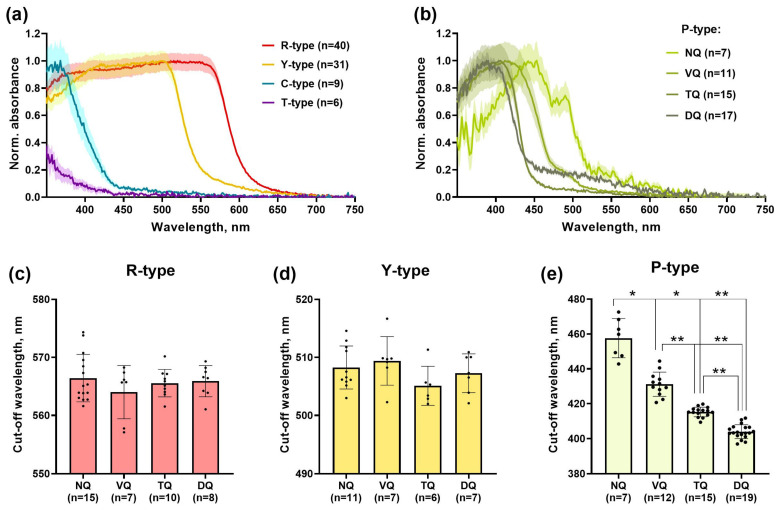
Spectral characteristics of ODs in European robin’s retina. (**a**) The mean normalized absorbance spectra of the R-, Y-, C- and T-type ODs of the European robin’s retina. (**b**) The mean normalized absorbance spectra of the P-type ODs in different retinal quadrants. The standard error of the mean is shown as the shaded intervals around each line. The values of cut-off wavelength (λ_cut_) calculated from preparations of different retinal quadrants for R- (**c**), Y- (**d**) and P-type (**e**) ODs. Data presented as mean ± SD. NQ, VQ, TQ, and DQ—nasal, ventral, temporal, and dorsal quadrants, respectively. *, ** Statistically significant difference according to the Brown–Forsythe ANOVA test with the post hoc Dunnett test for multiple comparisons (*p* < 0.05 and *p* < 0.0001, respectively). OD—oil droplet, *n*—total number of oil droplets scanned for each retinal quadrant. Since there were no significant differences in (λ_cut_) for R- and Y-type ODs between retinal quadrants, data from whole retinas were pooled and averaged for representation in panel (**a**).

The ODs are located in the apical part of the cones’ inner segments right under the outer segment which is a light-sensitive structure containing visual pigments and other phototransduction proteins. As they are located in the way of light falling onto the outer segment, they can act as small focusing lenses, and in the case of colored ODs, as long-pass cut-off filters [38,39]. Thus, ODs are characterized by their cut-off wavelength (λ_cut_), below which no light of shorter wavelengths is transmitted further to the cone outer segment (33). Such filtering reduces the spectral sensitivity overlapping between different cone types and improves the discriminability of individual wavelengths that may enhance the animal’s color vision [40]. We recorded the spectra of all five OD types in European robin’s retinal preparations using microspectrophotometry and calculated their λ_cut_ values. Even though it was not possible to distinguish between C- and T-type droplets by light microscopy, the spectral recordings allowed us to reveal two distinct populations of short-wavelength filtering ODs. The first one has λ_cut_ = 370 nm and was defined as C-type, while second one had only a small slope in near UV range (λ_cut_ < 330 nm) as expected for T-type (Figure 7a). We found only a small number of C- and T-type ODs in all retinal preparations, so we could not perform any topographic analysis of their spectra. On the other hand, higher density of R-, Y- and P-type droplets allowed us to compare them between retinal quadrants. Generally, their spectral properties were the same as for most species of birds studied to date [24,29,41], and we found no differences in λ_cut_ values between quadrants for R- and Y-type ODs (Figure 7a,c,d). However, P-type ODs located in double cones showed an interesting pattern of filtering characteristics: they varied significantly between different retinal parts, and the highest λ_cut_ was observed in the nasal quadrant (Figure 7b,e). The ventral part also contained the pale yellow ODs with long-wavelength-shifted λ_cut_, while in the dorsal quadrant it was about 400 nm, so they are expected to look almost transparent to the observer’s eye, like C- and T-type. Therefore, the microspectrophotometry confirmed the data obtained by light microscopy and provided an explanation for the unusual coloration of P-type ODs in the naso-ventral region—the shift in cut-off wavelength. All λ_cut_ values are presented in Table 3.

## 4. Discussion

### 4.1. Insight into Basic Properties of Magnetoreception from Electrophysiological Data

A large body of behavioral data suggests that the avian magnetic compass is located in the retina (for reviews, see [13,42]). In our previous studies, we tested the impact of changing magnetic field on visual signals in different bird species [20,21,22] and discovered small effects that presumably arise from the magnetoreception. Here, we report the extension of our electrophysiological study on European robins where we combined background illumination with brief flashes of the opposite color (blue flash + red background and vice versa) and changing of magnetic field direction. Surprisingly, from these light condition combinations we observed an effect of magnetic field direction only for responses to red flashes plus blue continuous background light, but not on responses to blue flashes plus red background light. As the molecular and cellular mechanisms of magnetoreception remain mostly unknown, we only could speculate with extreme caution about the properties underlying the effect we observed. The small size of the observed MF effect may have a biological explanation related to the features of the ex vivo ERG method, which provides the resultant signal from thousands of cells located in the tested quadrant. Most probably, magnetosensitive cells make up a quite small proportion of the retinal cells (for example, a small population of one type of cones), and the response from this population would be strongly diluted by the signal from the cells that do not possess a magnetoreceptive function. In addition, it points to the fact that ex vivo ERG recordings are not the optimal approach for further studying particular magnetoreception mechanisms. Our previous electrophysiological study of European robin’s retina [22] revealed that magnetosensitivity appears after stimulation by blue, but not by red, light. Moreover, high-intensity blue flashes also suppress magnetoreception, in agreement with behavioral data [5,43]. Our newest results show that magnetoreception can be observed in the condition of red flash if it is applied over a dim blue background illumination, providing additional proof for short wavelength-dependence of magnetic sense.

The absence of magnetosensitivity (or a response so small that it is completely diluted in the total retinal signal) under blue flash stimulation combined with continuous red background may seem contradictory. However, the intensities of blue flashes we used were the same as the ones defined as “bright” in our previous study [22], since stimulation by lower intensity could not induce any significant analyzable response from the retina under background light adaptation. According to our previous electrophysiological results, the red light does not seem to induce any magnetosensitivity, making the combination of bright blue flashes with red background functionally indistinguishable from the bright blue flashes applied in darkness [22]. Our electrophysiological data are consistent with numerous studies of avian orientation behavior and neuronal activity that support the notion that too high light intensity disrupts magnetoreception [5,43,44,45]. It also appears that a bright 10 ms flash provides too many photons for the sensory system, while the continuous background with about two orders of magnitude lower intensity (in photons/mm^2^/ms) does not suppress the magnetosensitivity. This result could be explained if we assume that a magnetoreceptive cell becomes almost or completely saturated after high light exposure and response to a certain external MF, being unable to react properly to further MF changes. Additionally, adaptation mechanisms could exist and prevent saturation under dim continuous illumination, resembling the light adaptation in photoreceptors (for review, see [46]). As photoactivation of cryptochrome molecules converts them into a magnetosensitive state [8], too many “sensory active” molecules may provide too much of a signal at once. This leads to “saturation” of magnetoreceptive cells—like after a brief but bright flash—and makes it temporarily insensitive to further stimulation by the rotation of the magnetic vector. On the other hand, if the photon flux is not too high, the adaptation mechanisms will keep the receptor “unsaturated” even if the illumination is continuous, enabling response to changes in MF. Since Cry4 was shown to be able to interact with cone-specific phototransduction proteins [17,18], one can assume that magneto- and photoreception share the same light-adaptation mechanisms.

### 4.2. Morphological Features of European Robin’s Nasal Retina: Possible Connection of Oil Droplets with Magnetoreceptive Function

Since the effect of changing MF on visual response was observed only in the nasal quadrant of European robin retina, together with our previous results [22] it gives reasonable support to the hypothesis that magnetoreceptive cells are localized in this particular area. Therefore, we decided to analyze different cone type distributions throughout the retina of this species in an attempt to find evidence of the nasal part’s exceptionality. Whereas we did not observe the differences that would exclusively highlight the nasal quadrant in distribution density, size or proportion of three analyzable cone types (R-, Y- and P-type ODs that correspond to LWS, MWS single cones and double cones, respectively), an interesting finding concerning P-type OD coloration was revealed: in most of the nasal quadrant and in a part of the ventral quadrant close to the nasal one, the P-type ODs had a well-marked greenish yellow color, while the same ODs in all remaining retinal parts had a much less intensive coloration. This feature, firstly discovered by crude visual examination of flat-mount preparations and under a light microscope, was subsequently confirmed by microspectrophotometry, showing that spectra of the P-type ODs had different cut-off wavelengths in different quadrants, with the longest values in nasal and ventral quadrants.

It should be noted that intraretinal variation in coloration and spectral transmittance characteristics of P-type ODs, particularly the long wavelength shift in λ_cut_ (about 60 nm) between ODs in dorsal and ventral areas, was described previously in other bird species. Three of them, the starling (*Sturnus vulgaris*, [47]), the blackbird (*Turdus merula*, [29]) and the wood thrush (*Hylocichla mustelina*, [48]), together with the European robin, are known to be migratory at least in some parts of their range. Most of other studied non-migratory passerines do not demonstrate such spectral differences in P-type ODs between retinal parts, with the exception of the white-headed munia (*Lonchura maja*), which shows about a 70 nm shift in λ_cut_ between the dorsal and the ventral areas [29,41,49,50]. Additionally, a well-known passerine long-distant migrant, the barn swallow (*Hirundo rustica*, [48]), does not show this pattern of P-type OD spectral distribution. Still, in none of the aforementioned studies was the nasal quadrant indicated as exceptional and only ODs from dorsal and ventral halves were compared.

The colored ODs serve as light-filtering structures, which affect the light received by magnetosensitive molecules (presumably cryptochromes) inside cones’ outer segments. As the spectral composition of this light would determine whether such molecules will be turned into the activated state, the ODs’ function should be tightly coupled with magnetoreception. Therefore, we could suggest that specific filtering characteristics of nasal P-type ODs might play a role in MF perception. On the other hand, we are aware that there might be no connection between them. For example, Hart et al. proposed that the function for more intensive coloration of ventral retina P-type ODs is to reduce of harmful short-wavelength radiation coming from the sky in the bird’s upper visual field [29]. Interestingly, it was hypothesized that ODs could act as detectors of the Earth’s MF themselves if they were liquid crystals containing magnetite particles [51]. However, we are not adherent to this idea since there is no evidence for magnetite-containing cell structures within the retina [52]. It is also important to mention that the P-type ODs of birds kept in captivity for a long time (more than four weeks) displayed λ_cut_ values shifted to shorter wavelengths typical for the dorsal quadrant, independently of their localization (see Supplementary Figure S5). This phenomenon is probably reflecting a dietary deficiency of particular carotenoids [53] and should also be taken into account in studies concentrating on the role of double cones in magnetoreception. In our research, all electrophysiological experiments and relevant microspectrophotometric measurements were performed within one month once birds were captured.

### 4.3. Nasal Retinal Double Cones as Potential Magnetoreceptors

Our results allow us to suggest that the nasal quadrant of the European robin’s retina exhibits a potential for magnetosensitivity in electroretinographic testing and, additionally, contains double cones with extremely long-wavelength-shifted ODs as light filters. The fact that we discovered the double cones to be a distinctive cell type in the potentially magnetoreceptive nasal quadrant matches with other studies. The putative primary magnetoreceptive molecule, Cry4, was found to localize inside the European robin’s double cones’ outer segments [12], and these cells were shown to form a regular mosaic that is necessary for precise MF direction sensitivity [54]. The individual double cone was proposed as a magnetoreceptor in several studies [13,19], and the most current model does not impose any particular restrictions on the spectral properties of the OD but demands both principal and accessory cones droplets to transmit the incident light independently of its intensity and polarization. Still, our results allow us to assume a magnetoreceptive function for nasal retinal double cones containing ODs that absorb almost all short-wavelength light required for cryptochrome activation. If both principal and accessory members would carry this type of droplet, the Cry4 molecules inside the double cones’ outer segments would receive an extremely low amount of light, making them unlikely to be magnetosensitive. This problem leads some researchers to doubt that double cones are MF receptors and propose the UVS cones associated with transparent ODs and expressing Cry1a as more promising candidates [55].

**Figure 8:**
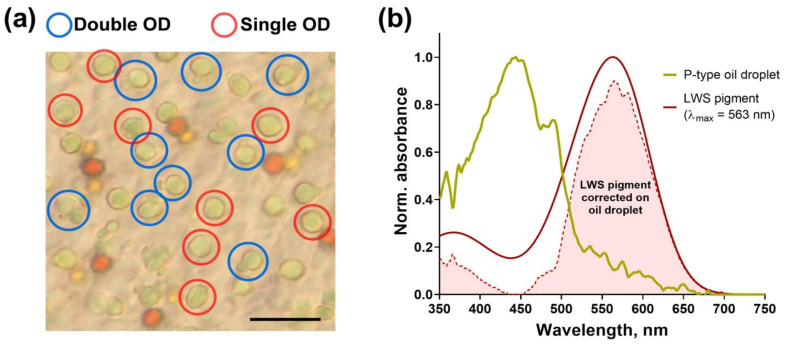
Double cones in the European robin’s retina. (**a**) Light microscopy images show two types of P-type ODs: single droplets (in red circles) and the ones tightly coupled with a small droplet of the same color (in blue circles), which is supposed to refer to the accessory cone. Scale bar = 10 µm. (**b**) Despite long-wavelength-shifted P-type ODs from nasal quadrant effectively blocking the short-wavelength incident light from reaching the cone outer segment, it leaves the LWS visual pigment spectrum mostly unaltered. The OD spectrum is the average from all nasal P-type droplets recorded (yellow solid line, *n* = 7), smoothed with 7-point moving average filter. LWS pigment spectrum (red solid line) was calculated according to Govardovskii et al. template [34] with λmax = 563 nm taken from the related species, the starling [47]. Red-filled area represents the corrected LWS pigment spectrum, calculated as overlap between OD transmittance and original LWS spectrum. OD—oil droplet.

Morphological data indicate that in some migratory passerine species, only the principal member of the double cone contains an OD, while the accessory one lacks it [29,47]. In the case of the European robin, we found with light microscopy a large number of the P-type droplets to be paired with a small, but similarly colored OD, obviously from the accessory cone. However, for some P-type ODs we could not observe a paired droplet, which could either be caused by disturbances in cell alignment during retinal preparation or imply the absence of ODs in accessory members (Figure 8a). Lacking the OD, these accessory cones will receive full-spectrum light, enough to activate both visual pigment and cryptochrome. Moreover, the data on the regularity of double cones’ orientation in the European robin’s retina suggest them to be able to form magnetosensitive pairs of neighboring cells with no need to compare signals between principal and accessory cones [54].

We suggest that in the European robin’s nasal retina at least some of the double cones contain ODs with long-wavelength-shifted λ_cut_ in the principal member only. The yellowish droplet is supposed to effectively block most of the short-wavelength light on its way to the outer segment though it transmits the yellow–red part of the visible spectrum without any significant intensity reduction. Calculation of spectra overlap between P-type OD transmittance and LWS visual pigment expressed in double cones shows that the latter’s main sensitivity peak is almost unaltered (Figure 8b). Since we could not perform measurements of the European robin’s visual pigment’s spectra due to small size and low absorbance of its cones’ outer segments (however, we were able to record the rod visual pigment spectrum, see Supplementary Figure S6), we used the data on a related passerine species, the starling [47]. Therefore, the P-type OD can function as a blocking structure for magnetosensitivity in the principal member of the double cone, leaving it as a typical photoreceptor while the OD-less accessory member would be able to react to an external MF.

## Figures and Tables

**Table 1:** Mean and standard deviation of the oil droplet density in the central and peripheral regions of four quadrants analyzed from retinas of five different European robins. NQ, VQ, TQ, and DQ—nasal, ventral, temporal, and dorsal quadrants, respectively; OD—oil droplet.

Quadrant and Region of the Retina	Type of OD, Density/mm^2^		
	R-Type	Y-Type	P-Type
Central region of NQ	2495 ± 1095	2583 ± 1375	16,059 ± 9277
Peripheral region of NQ	1878 ± 982	2297 ± 1388	12,433 ± 7976
Central region of VQ	1861 ± 919	2295 ± 1083	12,412 ± 6192
Peripheral region of VQ	962 ± 291	1130 ± 361	5628 ± 2126
Central region of TQ	2458 ± 1102	3023 ± 1578	19,289 ± 11,102
Peripheral region of TQ	1178 ± 827	1364 ± 864	7634 ± 4461
Central region of DQ	2878 ± 1211	3630 ± 1560	23,250 ± 8788
Peripheral region of DQ	2283 ± 1277	2771 ± 1593	19,376 ± 12,940

**Table 2:** Mean and standard deviation of the oil droplet diameters in the central and peripheral regions of four quadrants analyzed from retinas of five different European robins. NQ, VQ, TQ, and DQ—nasal, ventral, temporal, and dorsal quadrants, respectively; OD—oil droplet.

Quadrant and Region of the Retina	Type of OD, Diameter, µm		
	R-Type	Y-Type	P-Type
Central region of NQ	2.6 ± 0.3	1.8 ± 0.2	2.1 ± 0.4
Peripheral region of NQ	2.9 ± 0.3	2.2 ± 0.2	2.8 ± 0.3
Central region of VQ	2.7 ± 0.3	1.9 ± 0.3	2.3 ± 0.5
Peripheral region of VQ	3.2 ± 0.3	2.5 ± 0.3	3.3 ± 0.4
Central region of TQ	2.7 ± 0.4	2.0 ± 0.4	2.0 ± 0.3
Peripheral region of TQ	3.1 ± 0.3	2.2 ± 0.3	3.3 ± 0.6
Central region of DQ	2.5 ± 0.1	1.7 ± 0.2	1.9 ± 0.2
Peripheral region of DQ	2.7 ± 0.2	1.9 ± 0.2	2.5 ± 0.5

**Table 3:** Means and standard deviations of the oil droplets’ λ_cut_ in four quadrants analyzed from European robin’s retina. NQ, VQ, TQ, and DQ—nasal, ventral, temporal, and dorsal quadrants, respectively; OD—oil droplet.

Quadrant of the Retina	λ_cut_ of OD Type, nm				
	R-Type	Y-Type	P-Type	C-Type *	T-Type *
NQ	566.4 ± 4.1	508.2 ± 3.7	457.6 ± 11.3	370.06 ± 4.04	<330 nm
VQ	564.0 ± 4.6	509.4 ± 4.2	431.2 ± 6.9		
TQ	565.6 ± 2.4	505.1 ± 3.4	415.2 ± 2.8		
DQ	565.9 ± 2.7	507.3 ± 3.3	404.0 ± 3.9		

* ODs of these types were microspectrophotometrically analyzed without reference to a certain quadrant due to their rarity of occurrence.

## Data Availability

The data reported in this study are available upon request from the corresponding author and are not available to the public because of their size.

## Supplementary Materials

The following are available online at https://www.mdpi.com/article/10.3390/cells11193056/s1, Figure S1: Emission spectra of blue and red LEDs used for light stimulation of retinal samples; Figure S2: Effect of MF direction on the kinetics of the main components of ERG response, a-wave and b-wave, Figure S3; Graphical representation of the eight-direction distribution of the oil droplets’ spatial density, Figure S4: Graphical representation of the oil droplet percentage found in the four quadrants of the retina, Figure S5: The shift in cut-off wavelength observed in European robin’s P-type oil droplets after being kept in captivity for a long time, Figure S6: The absorbance spectrum of European robin’s rod photoreceptor recorded from isolated outer segments with microspectrophotometer, Table S1: Mean and standard deviation of the oil droplet percentage in the central and peripheral regions of four quadrants analyzed from retinas of five different European robins.
