# Supplementary material for: Magnetoreceptory Function of European Robin Retina: Electrophysiological and Morphological Non-Homogeneity
Source: Cells. 2022 Sep 29;11(19):3056. doi: 10.3390/cells11193056 (PMC9564291; doi:10.3390/cells11193056)
Supplement: Supplementary file 1 [file cells-11-03056-s001.zip › cells-1856454-supplementary.pdf]

cells MDPI

Supplementary Materials

Magnetoreceptory Function of European Robin Retina: Electrophysiological and Morphological Non-Homogeneity

Alexander Yu. Rotov [1], Arsenii A. Goriachenkov [1], Roman V. Cherbunin [1,2], Michael L. Firsov [1], Nikita Chernetsov [1,2,3] and Luba A. Astakhova [1,*]

[1] Laboratory of evolution of the sense organs, Sechenov Institute of Evolutionary Physiology and Biochemistry RAS, 194223 St Petersburg, Russia
[2] Spin Optics Laboratory, Physics Faculty, St.-Petersburg State University, 198504 Ulyanovskaya 1, St.-Petersburg, Russia
[3] Department of Vertebrate Zoology, Biological Faculty, St. Petersburg State University, 199034 St. Petersburg, Russia
[4] Ornithology Lab, Zoological Institute RAS, 199034 St. Petersburg, Russia
* Correspondence: lubkins@yandex.ru

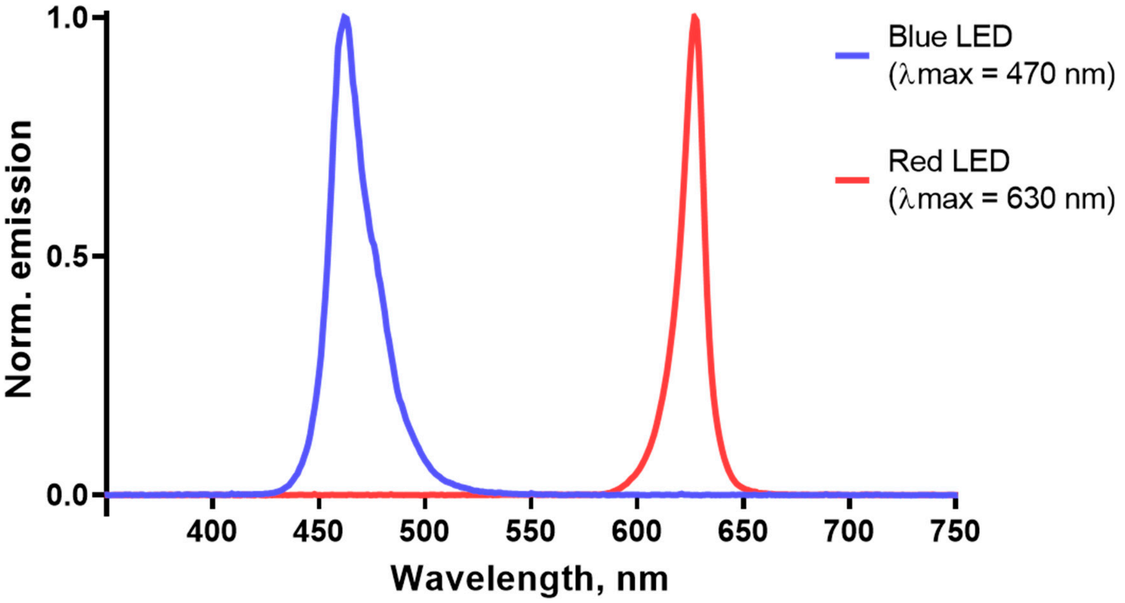

Figure S1. The emission spectra of two LEDs used for light stimulation in the electrophysiological setup. Spectral measurements were performed using USB4000 spectrometer (Ocean Optics, Orlando, FL USA). Intensities of LEDs were measuredat the same spot where the retina samples were located with an OPT-301 optosensor (Burr-Brown Corporation, Tucson. Arisona USA).

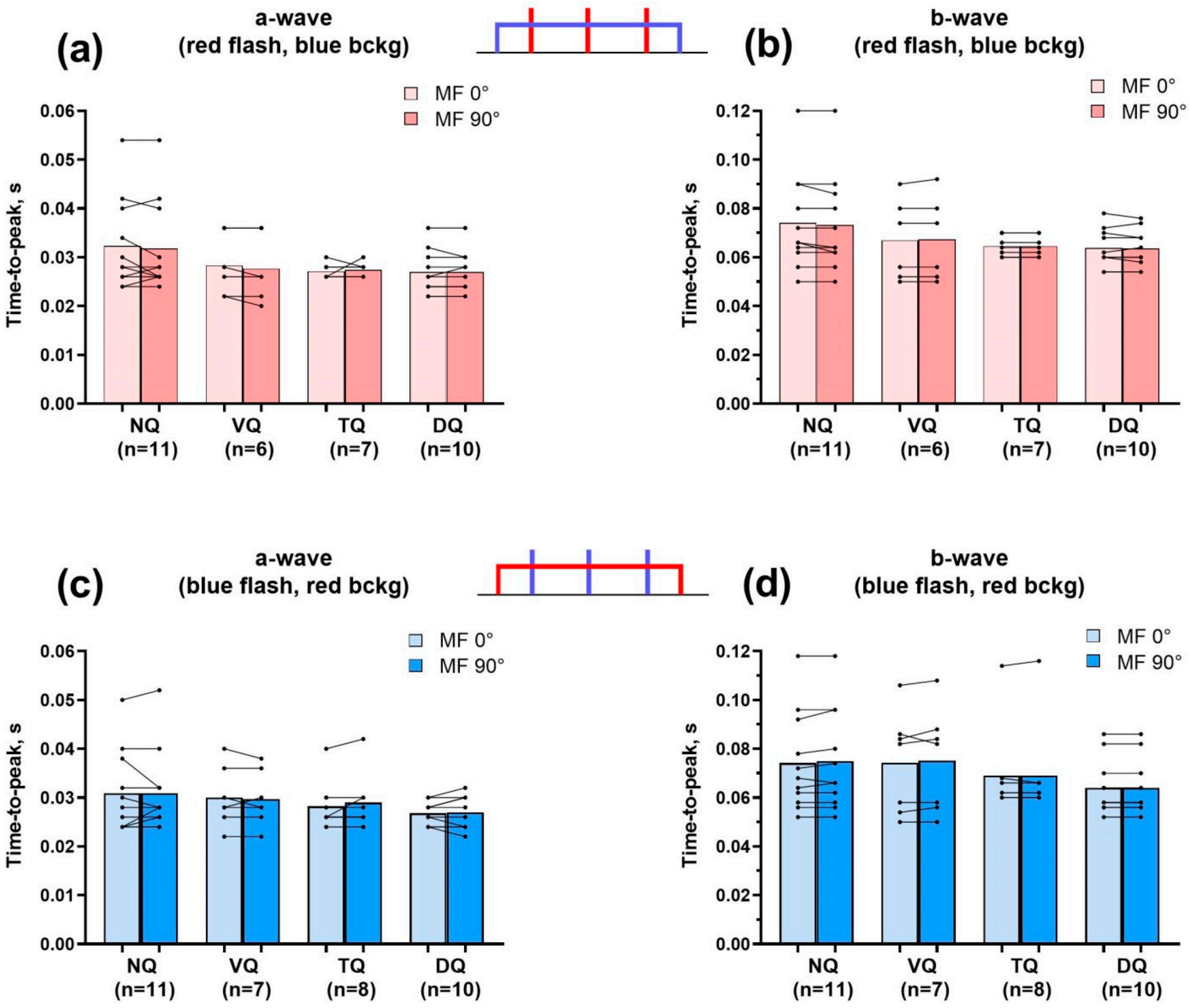

Figure S2. Effect of MF direction on the kinetics of the main components of European robin's ERG-response, a-wave (**a**,**c**) and b-wave (**b**,**d**). Bars show the a- or b-waves' time-to-peak intervals for responses recorded under particular MF direction (0° or 90° with respect to retinal plane) after stimulation with red 10-ms flashes under dim blue continuous background light (**a**,**b**) or blue 10-ms flashes under dim red continuous background light (**c**,**d**). In each panel, the results for the nasal (NQ), dorsal (DQ), temporal (TQ) and ventral (VQ) quadrants are presented separately.No statistically significant differences were detected by a Wilcoxon test for paired samples with the Holm–Bonferroni correction. Bars represent the means of corresponding samples, n – number of retinal preparations in each group.

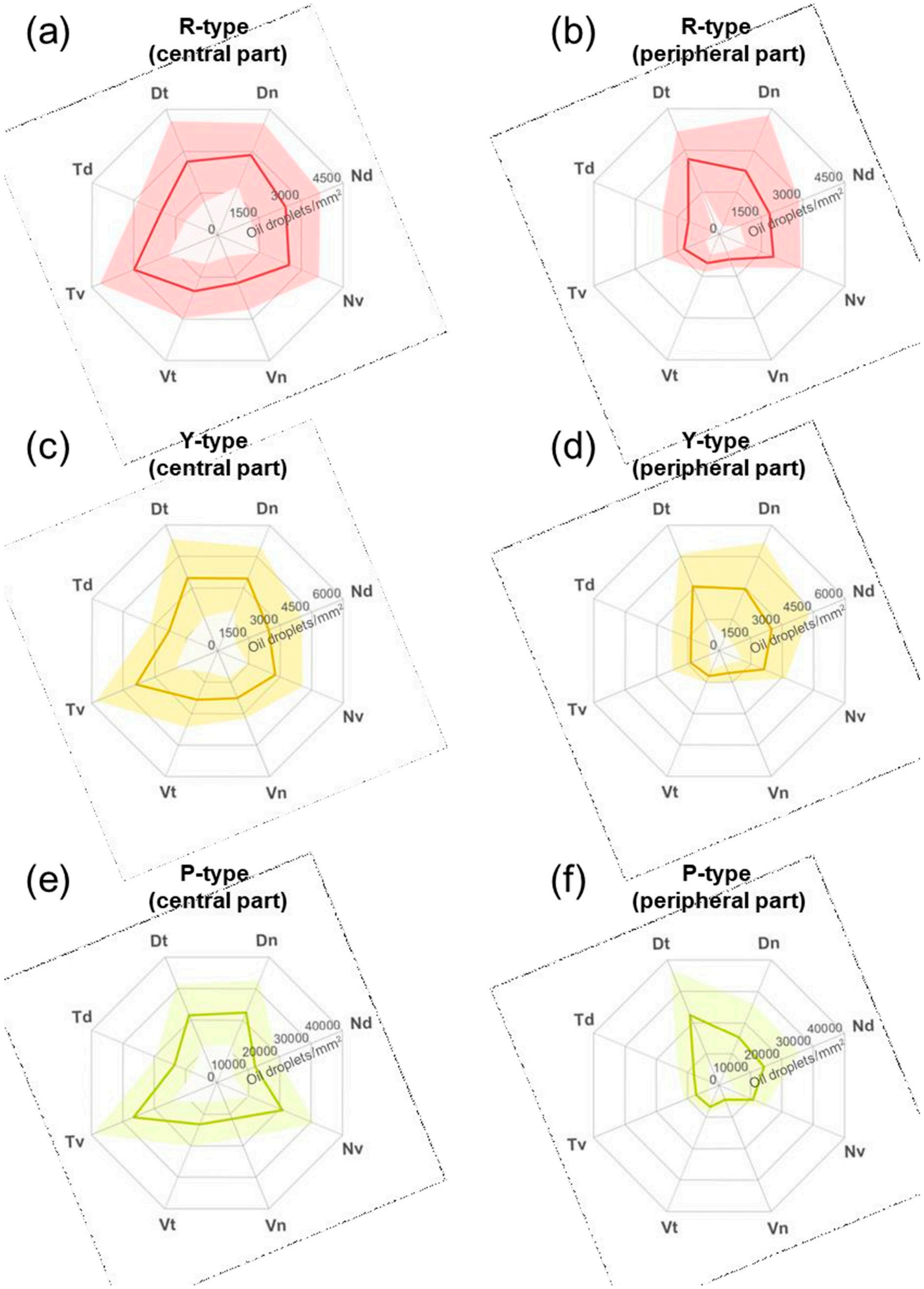

Figure S3. Eight-direction graphical representation of the three types oil droplets density distribution across the European robin's retina. Solid lines are the average density, while dim-colored areas represent SD. Capital letters N, V, T and D mean the nasal, ventral, temporal or dorsal quadrants, respectively to which the area belongs. Lowercase letters refer to the closest neighboring quadrant

to the particular area (for example, Dn means area from dorsal quadrant, close to the nasal one). The number of analyzed retinal preparations was 4–8 for each area.

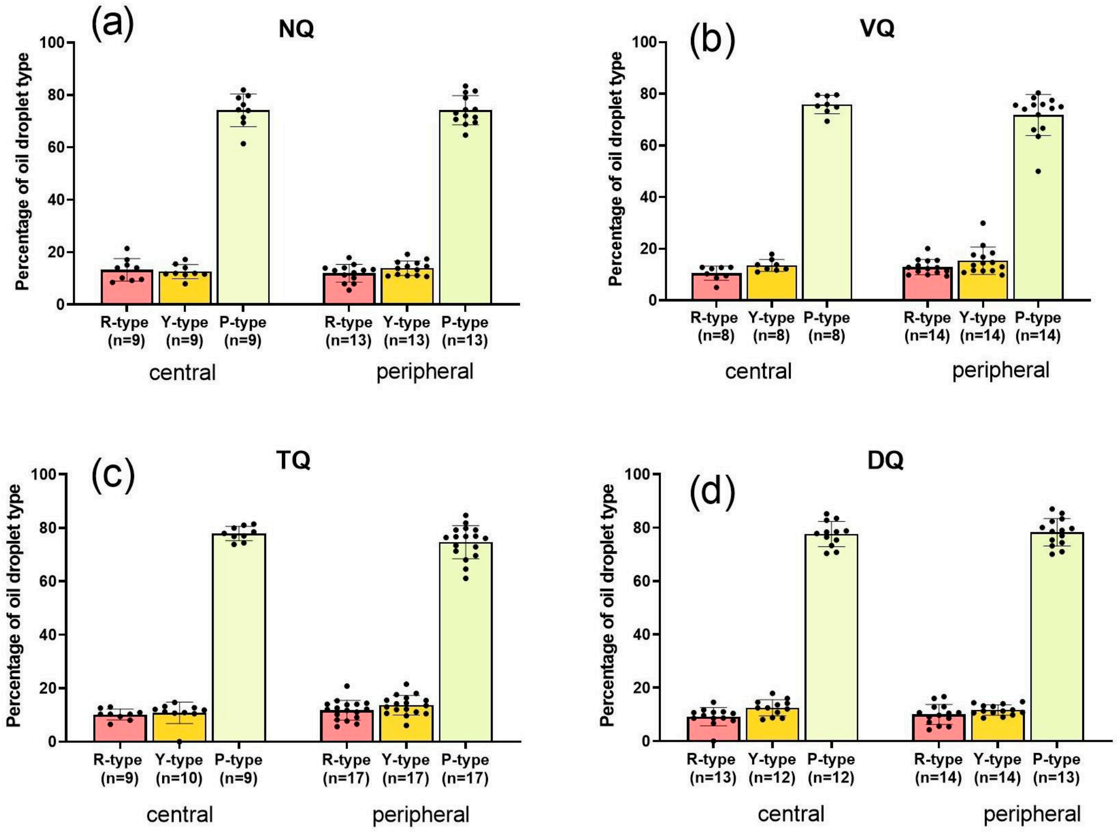

Figure S4. Graphical representation of the oil droplets percentage found in the four quadrants of the European robin'sretina. Data presented as mean ± SD. NQ, VQ, TQ and DQ – nasal, ventral, temporal and dorsal quadrants, respectively. No statistically significant differences were found between retinal quadrants, neither in central nor in peripheral parts. Value n – number of analyzed retinal preparations for each area.

Table S1. Mean and standard deviation of the oil droplet percentage* in the central and peripheral regions of four quadrants analyzed from retinas of five different European robins. NQ, VQ, TQ and DQ – nasal, ventral, temporal and dorsal quadrants, respectively; OD – oil droplet.

**Quadrant and Region of the Retina**	**R-type**	**Y-type**	**P-type**
Central region of NQ	13.2 ± 4.3	12.6 ± 2.7	74.2 ± 6.2
Peripheral region of NQ	12.0 ± 3.4	13.9 ± 2.8	74.2 ± 5.6
Central region of VQ	10.5 ± 2.8	13.6 ± 2.3	75.9 ± 3.5
Peripheral region of VQ	12.9 ± 3.0	15.4 ± 5.3	71.8 ± 8.0
Central region of TQ	10.1 ± 2.0	10.8 ± 4.0	77.9 ± 2.7
Peripheral region of TQ	11.7 ± 3.7	13.6 ± 3.7	74.6 ± 6.2
Central region of DQ	9.2 ± 3.4	12.5 ± 3.0	77.6 ± 4.7
Peripheral region of DQ	10.0 ± 3.7	11.7 ± 1.9	78.3 ± 5.2

*In the present study we could not take account ODs of C- and T-types, so the percentage is calculated as proportionof the sum of three accountable types (R, Y and P).

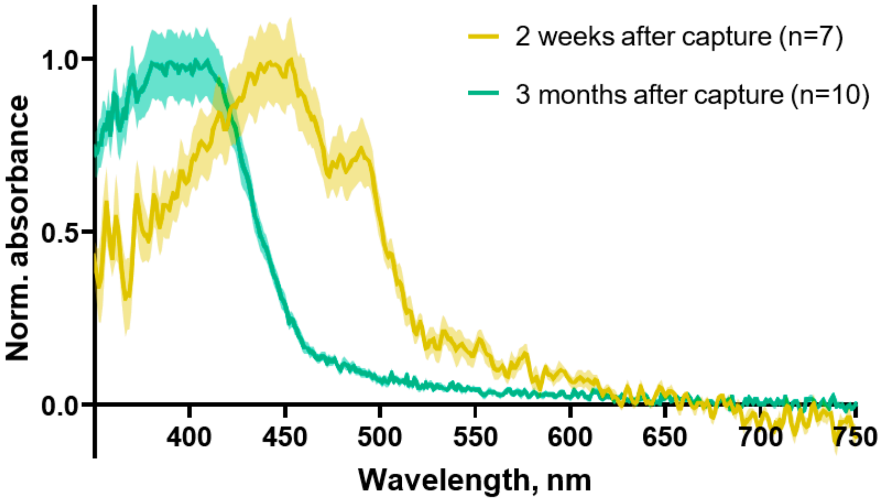

Figure S5. The shift of cut-off wavelength observed in European robin's P-type oil droplets after being kept in captivity for a long time. This phenomenon has been reported previously (see [1–3]) and is supposed to reflect a dietary deficiency of particular carotenoids. Yellow line represents the normalized averaged spectrum of oil droplets from nasal quadrant of the bird sacrificed within 2 weeks after capture (average λ_{cut} = 458 nm). Green line represents the same spectrum but for nasal quadrant oil droplets of the bird sacrificed after 3 months in captivity (average λ_{cut} = 411 nm). Dim-colored areas around lines represent the SEM. All spectra reached the finalized statistical analysis (see main text) were recorded within 3 weeks since birds' capture.

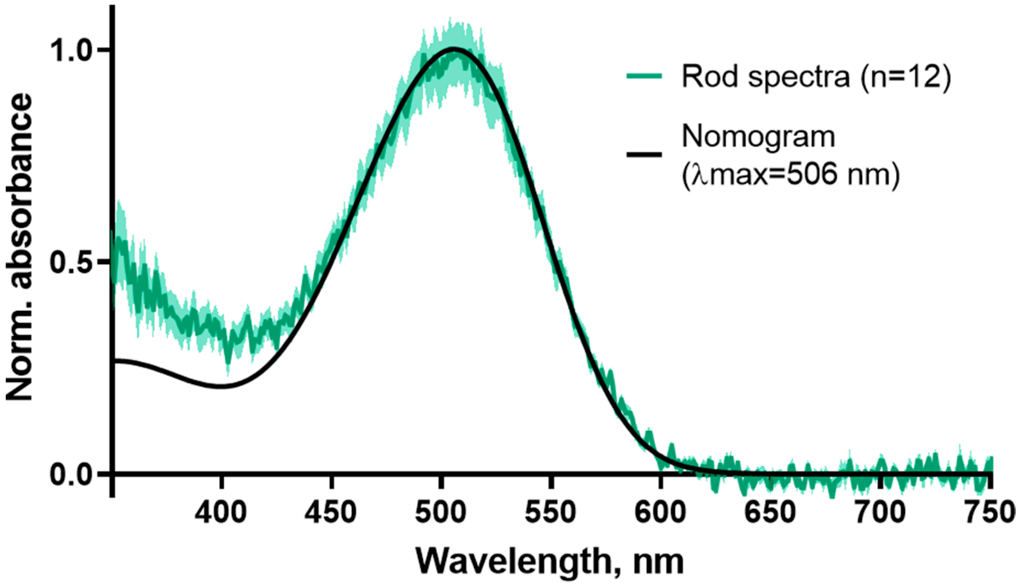

Figure S6. The absorbance spectrum of European robin's rod photoreceptor recorded from isolated outer segments with microspectrophotometer. Green line is the normalized spectrum averaged from 12 rods, dim-colored areaaround it represents the SEM. Black solid line is the spectrum fit by standard nomogram [4] with peak absorbance at 506 nm, typical for all studied avian species [5].

References

1. Goldsmith, T.H.; Collins, J.S.; Licht, S. The cone oil droplets of avian retinas. *Vis. Res.* **1984**, *24*, 1661–1671.
2. Hart, N.S.; Partridge, J.C.; Cuthill, I.C. Visual pigments, oil droplets and cone photoreceptor distribution in the European starling (*Sturnus vulgaris*). *J. Exp. Biol.* **1998**, *201*, 1433–1446.
3. Knott, B.; Berg, M.L.; Morgan, E.R.; Buchanan, K.L.; Bowmaker, J.K.; Bennett, A.T. Avian retinal oil droplets: Dietary manipulation of colour vision? *Proc. R. Soc. B* **2010**, *277*, 953–962.

4. Govardovskii, V.I.; Fyhrquist, N.; Reuter, T.; Kuzmin, D.G.; Donner, K. In search of the visual pigment template. *Vis. Neurosci.* **2000**, *17*, 509–528.
5. Hart, N.S. The visual ecology of avian photoreceptors. *Prog. Retin. Eye Res.* **2001**, *20*, 675–703.
